# Identification of BRAF, CCND1, and MYC mutations in a patient with multiple primary malignant tumors: a case report and review of the literature

**DOI:** 10.1186/s12957-023-03036-3

**Published:** 2023-05-24

**Authors:** Zheyu Liu, Cheng Jin, Yi Zhang, Yongquan Jiang, Jingshuo Wang, Luying Zheng

**Affiliations:** 1 Shanghai Jiao Tong University School of Medicine, Shanghai, 200025 People’s Republic of China; 2 Department of Otorhinolaryngology, Renji Hospital, School of Medicine, Shanghai Jiao Tong University, Shanghai, 200127 People’s Republic of China; 3 Department of Pathology, Renji Hospital, School of Medicine, Shanghai Jiao Tong University, Shanghai, 200127 People’s Republic of China

**Keywords:** Multiple primary malignant tumors, Genetic linkage, Malignant melanoma, Papillary thyroid carcinoma, Clear-cell renal cell carcinoma

## Abstract

**Background:**

Multiple primary malignant tumors (MPMTs), usually associated with worse malignant behavior and prognosis comparing to a single primary tumor, and have recently been found to have an increasing incidence globally. However, the pathogenesis of MPMTs remains to be clarified. Here, we report a unique case of the coexistence of malignant melanoma (MM), papillary thyroid carcinoma (PTC), and clear-cell renal cell carcinoma (ccRCC) along with our perceptions on its pathogenesis.

**Case presentation:**

The case reported is of a 59-year-old male patient with unilateral nasal obstruction as well as a renal occupying lesion. Positron emission tomography-computed tomography (PET-CT) revealed a palpable mass of 32 × 30 mm on the posterior and left walls of the nasopharynx. In addition, an isodense nodule was observed in the right superior renal pole, approximately 25 mm in diameter, as well as a slightly hypodense shadow in the right leaf of the thyroid, approximately 13 mm in diameter. Nasal endoscopy and magnetic resonance imaging (MRI) confirmed the existence of a nasopharyngeal neoplasm. Afterward, biopsies of the nasopharyngeal neoplasm, thyroid gland and kidney were performed, and the patient was diagnosed with MM, PTC, and ccRCC according to the pathological and immunohistochemical results. Moreover, mutation of BRAF^V600E^ was detected in bilateral thyroid tissues, and amplification of both CCND1 and MYC oncogenes were detected in the nasopharyngeal melanoma. After chemotherapy, the patient is now in good overall condition.

**Conclusions:**

This is the first reported case of a patient with the co-existence of MM, PTC and ccRCC undergoing chemotherapy with a favorable prognosis. Herein, we suggest that such a combination may be non-random, as for mutation of BRAF^V600E^ might account for the co-occurrence of PTC and MM, while mutations of CCND1 and MYC cause the coexistence of MM and ccRCC. This finding may provide valuable guidance on the diagnosis and treatment of such disease, as well as the prevention of developing a second or third tumor for patients with a single primary.

## Background

Multiple primary malignant tumors (MPMTs) are defined as two or more malignant tumors with various pathogenic origins detected simultaneously or successively in an individuality. Due to the time interval of diagnosis for the first and second primary tumors, MPMTs can be classified into two categories, synchronous (< 6 months) and metachronous (≥ 6 months) MPMTs [1]. The Warren and Gates criteria [2] adapted for MPMT diagnosis establishes that each tumor must be confirmed to be malignant, each tumor must be distinct, and all tumors must be primary rather than metastases of each other. The IARC criteria [3] added that tumors must also be located in different organs and share different histologic origins.

Despite the improvements in medical technology and advancements of clinical diagnosis and treatment for cancer, the incidence of MPMTs has been escalating. It has been revealed that within a follow-up of 20 years, the incidence of MPMTs in a cancer population varies between 2.4% and 8%. Vogt A et al. (2017) put forward that the burden of MPMTs has been rising over the last decade due to the aging problem [4]. However, most reports on MPMTs refer to two primary malignancies, and cases of three or more primary malignant tumors are extremely rare. As yet, the pathogenesis of this disease remains to be clarified. Hypotheses exist that endogenous, exogenous, hereditary, and therapeutic factors may be involved in the cause of MPMTs. Endogenous factors include immune status, susceptibility and endocrine. Both congenital and acquired deficiency in immune surveillance and immune defenses increases the possibility to develop a MPMT [5]. Exogenous factors are represented by personal lifestyle and environmental factors, such as smoking and alcohol, low physical activity, as well as long exposure to ultraviolet rays and industrial pollution. Genetic factors may significantly favor the development of MPMTs. Therapeutic factors, including carcinogenic radiation treatment and chemotherapy, may also increase the risk of developing MPMNs.

In this paper, we report on identification of BRAF^V600E^, CCND1, and MYC mutations in a patient with synchronous triple primary malignant neoplasms, including malignant melanoma (MM), papillary thyroid carcinoma (PTC), and clear-cell renal cell carcinoma (ccRCC). This combination, to the best of our knowledge, has never been previously reported in the literature, and it raised a series of diagnostic, etiological and therapeutic issues persuading us to carry out a critical review of the literature.

## Case presentation

### Diagnosis

On September 1, 2022, a 59-year-old male patient was admitted to the Renji Hospital, Shanghai Jiaotong University School of Medicine, because of unilateral nasal obstruction on the left side accompanied with epistaxis for more than 2 months. Besides, a renal occupying lesion was detected 1 week prior. He did not complain of any other discomfort and did not report any relevant family history.

**Fig. 1:**
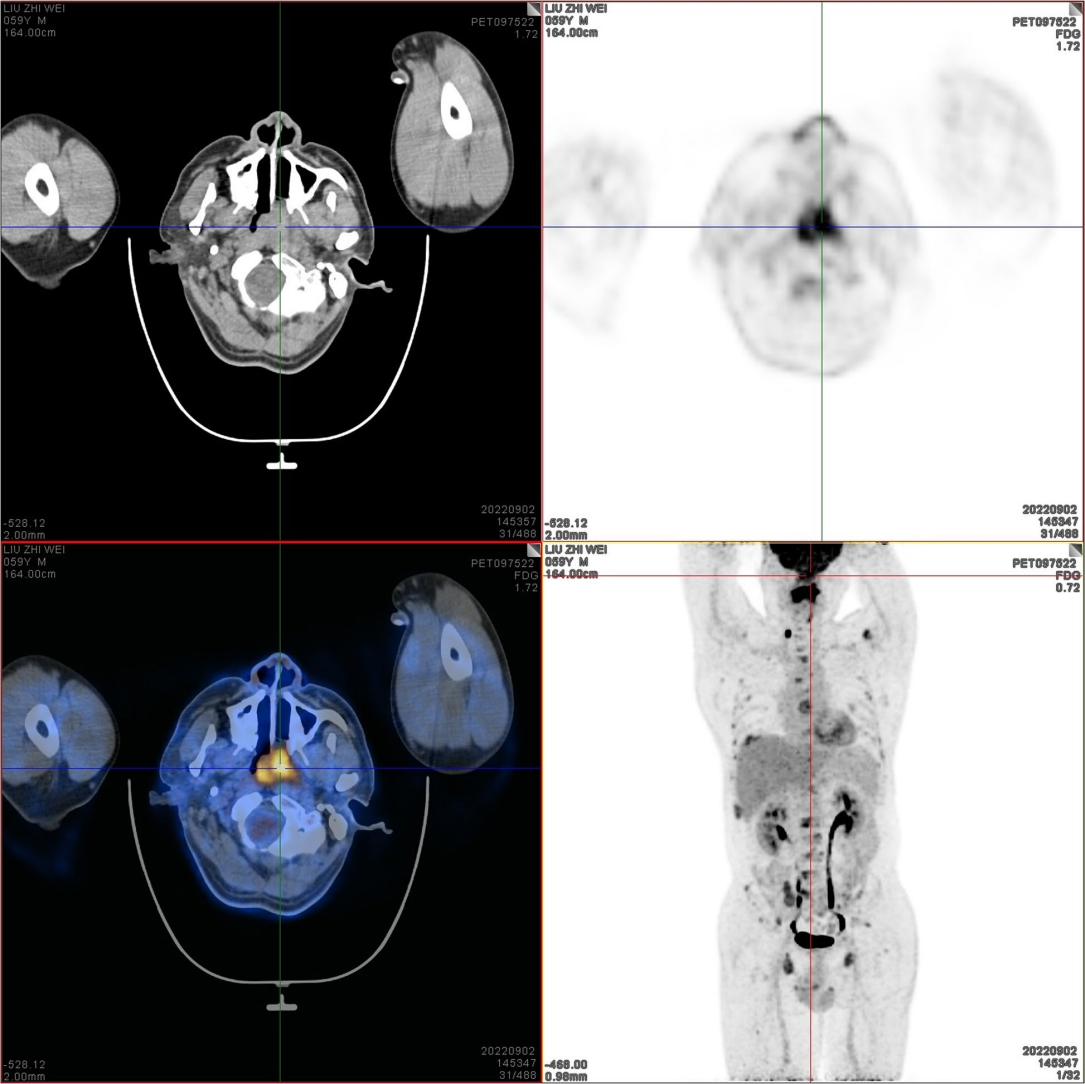
PET-CT scan findings of the loss of left pharyngeal recess and a palpable mass formed by incrassated soft tissue on posterior and left walls of the nasopharynx, of which the FDG metabolism increased

**Fig. 2:**
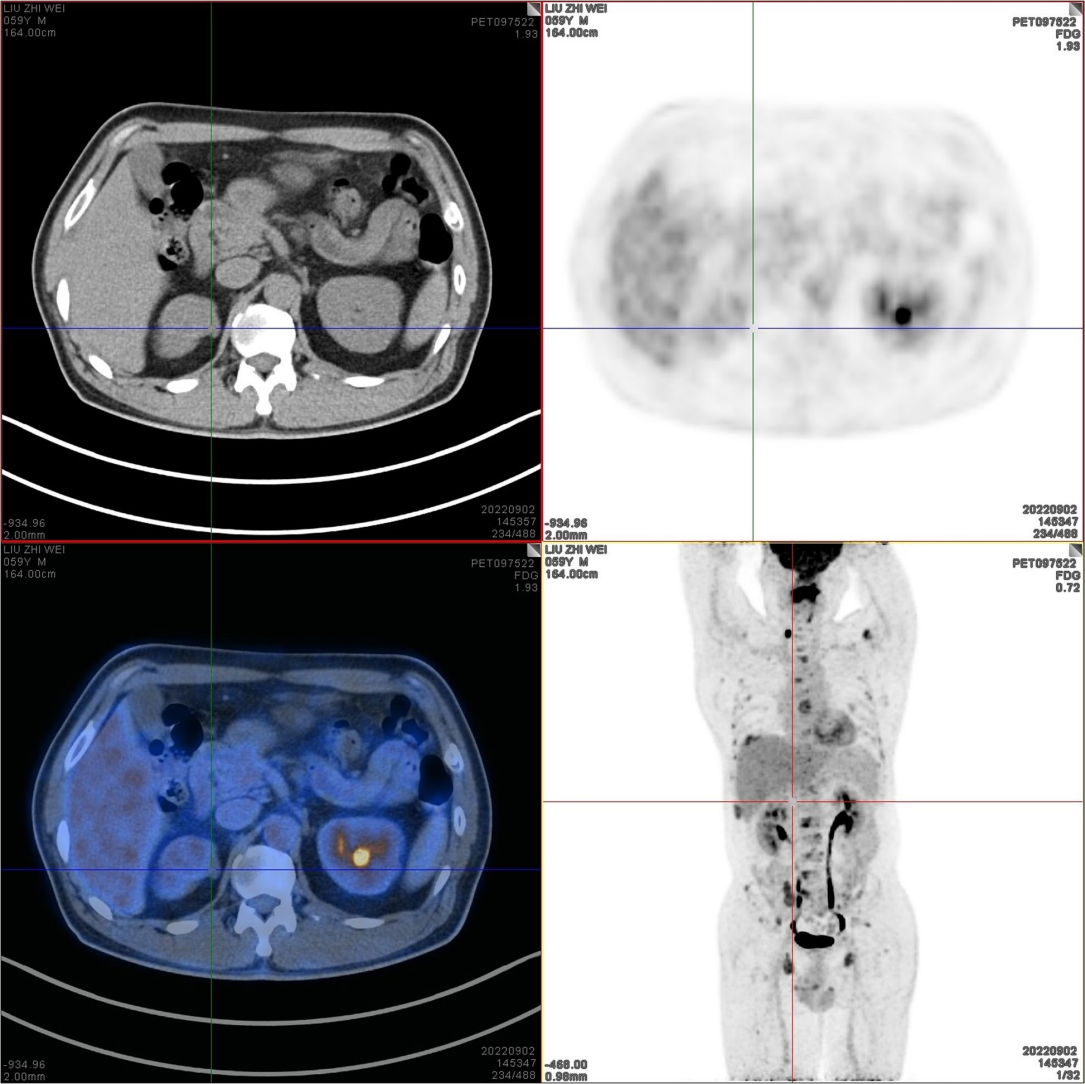
PET-CT scan findings of an isodense nodule in the right superior renal pole, approximately 25 mm in diameter

**Fig. 3:**
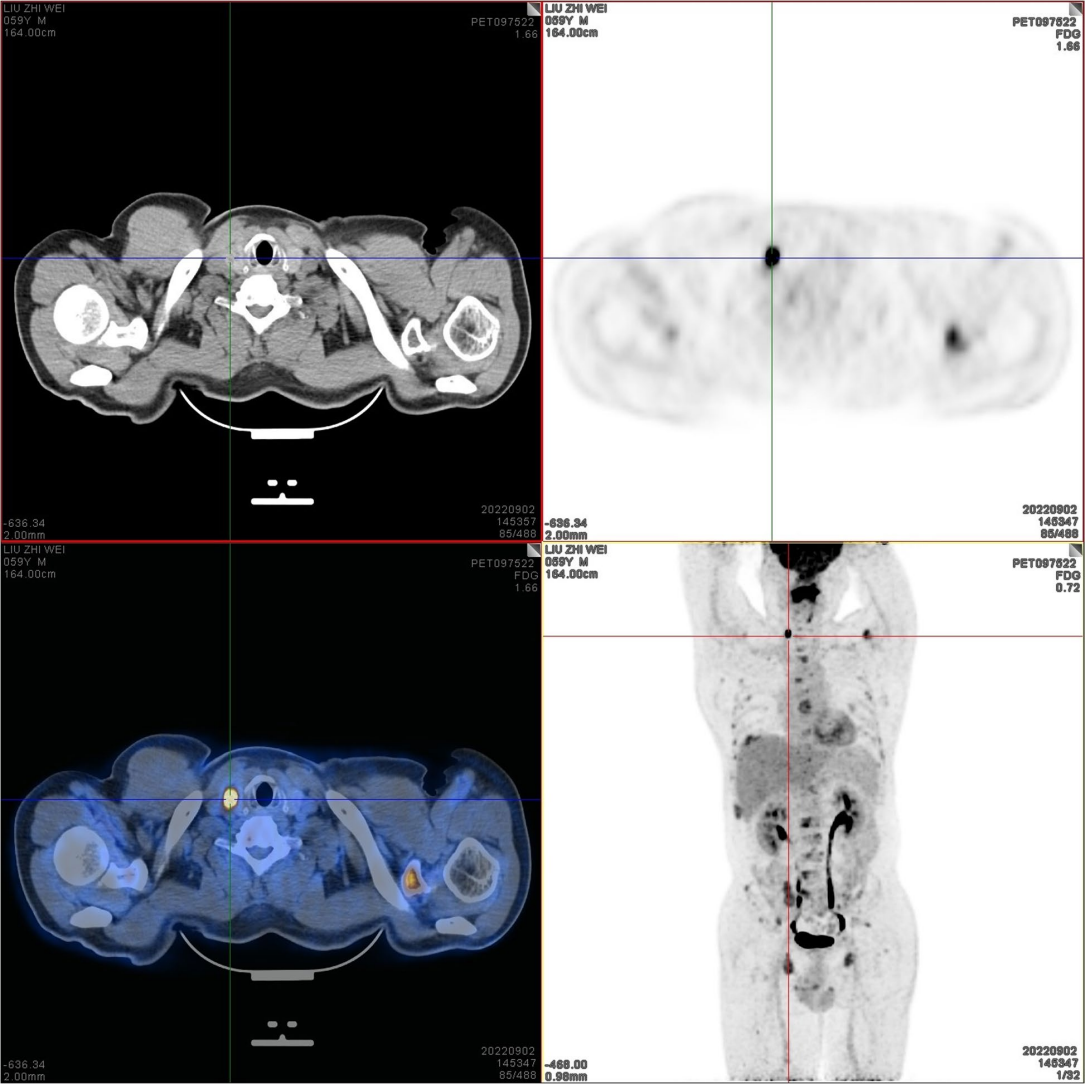
A slightly hypodense shadow in the right leaf of the thyroid was revealed

**Fig. 4:**
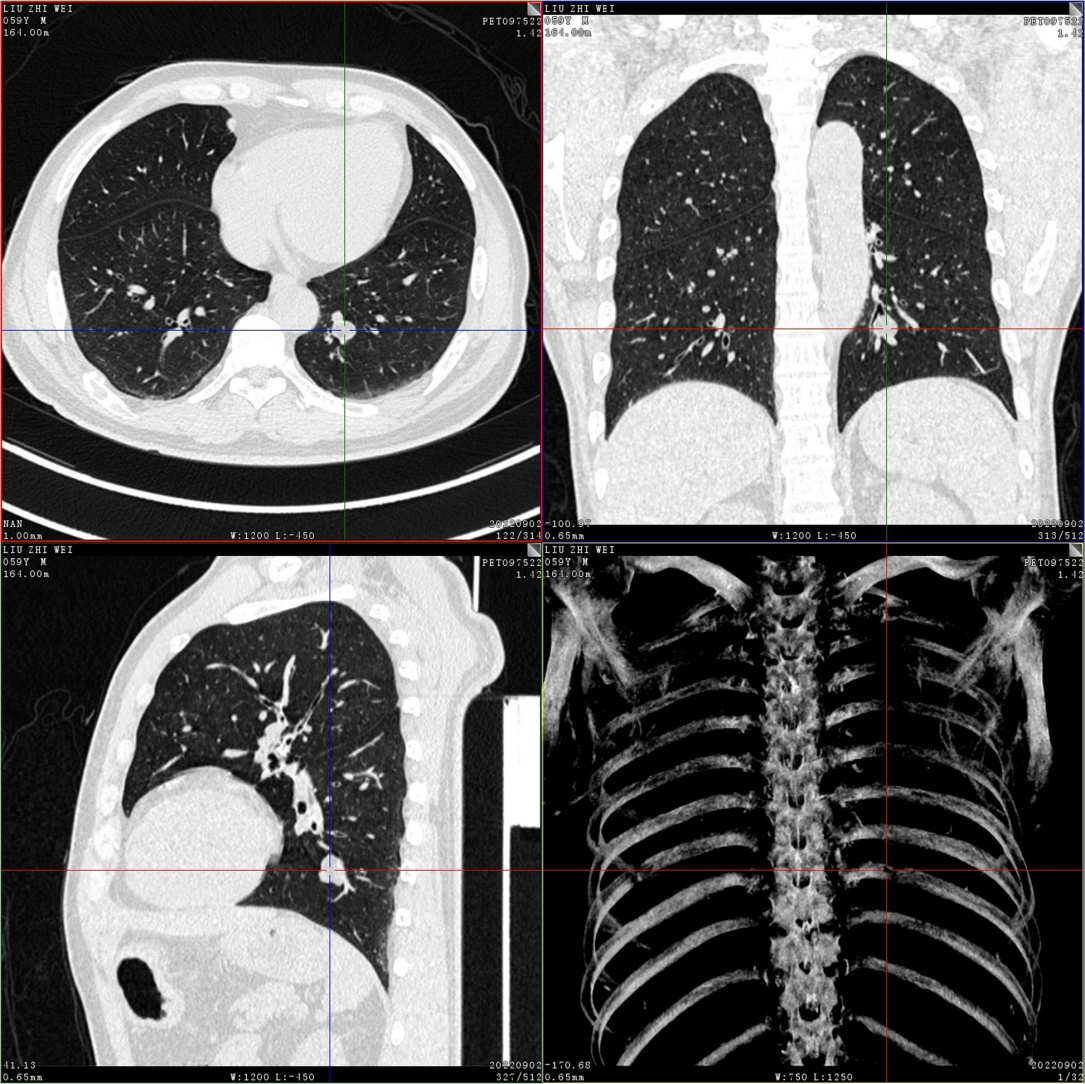
A nodule in the inferior lobe of left lung, 12 × 10 mm in size

**Fig. 5:**
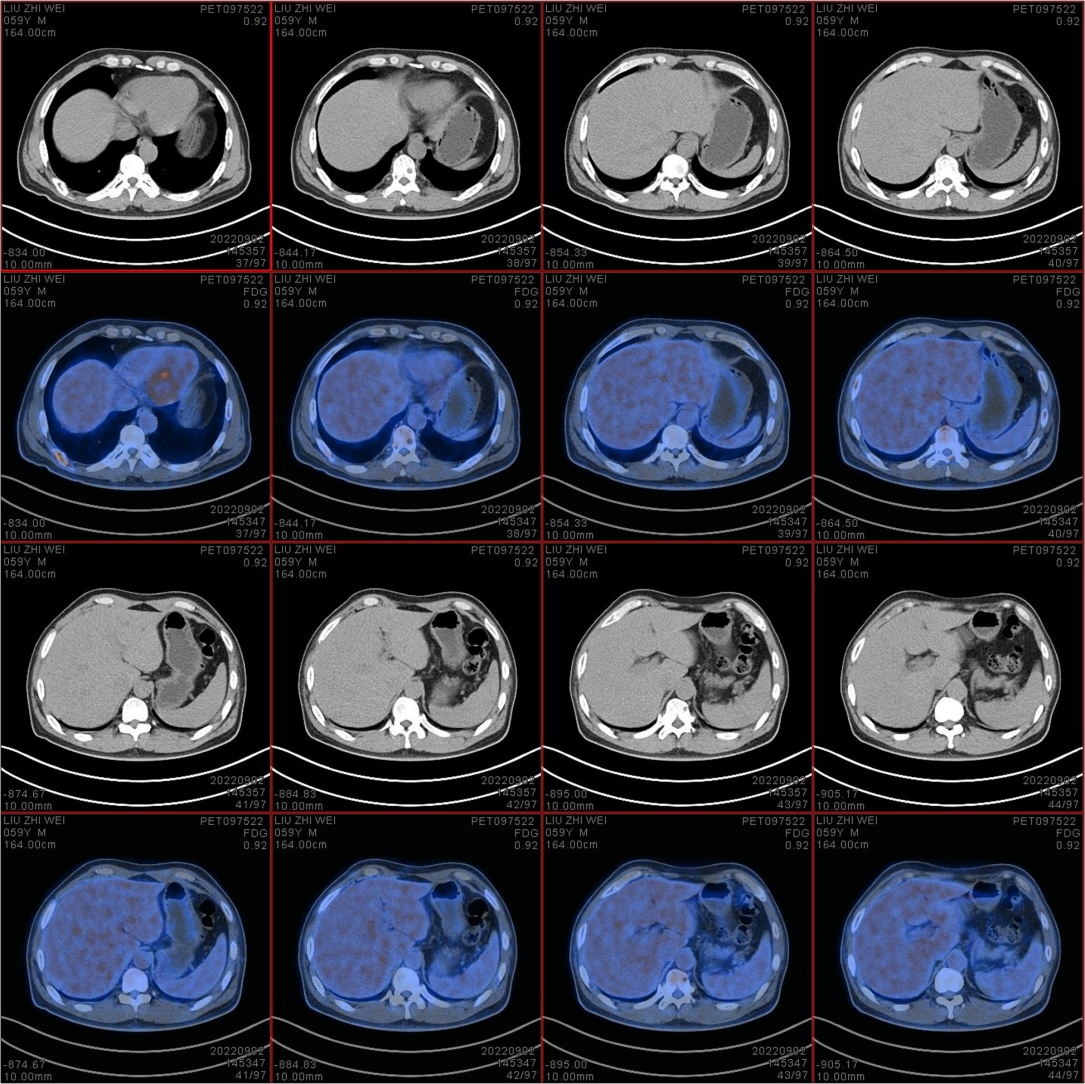
Multiple patchy shadows with low density in the liver

**Fig. 6:**
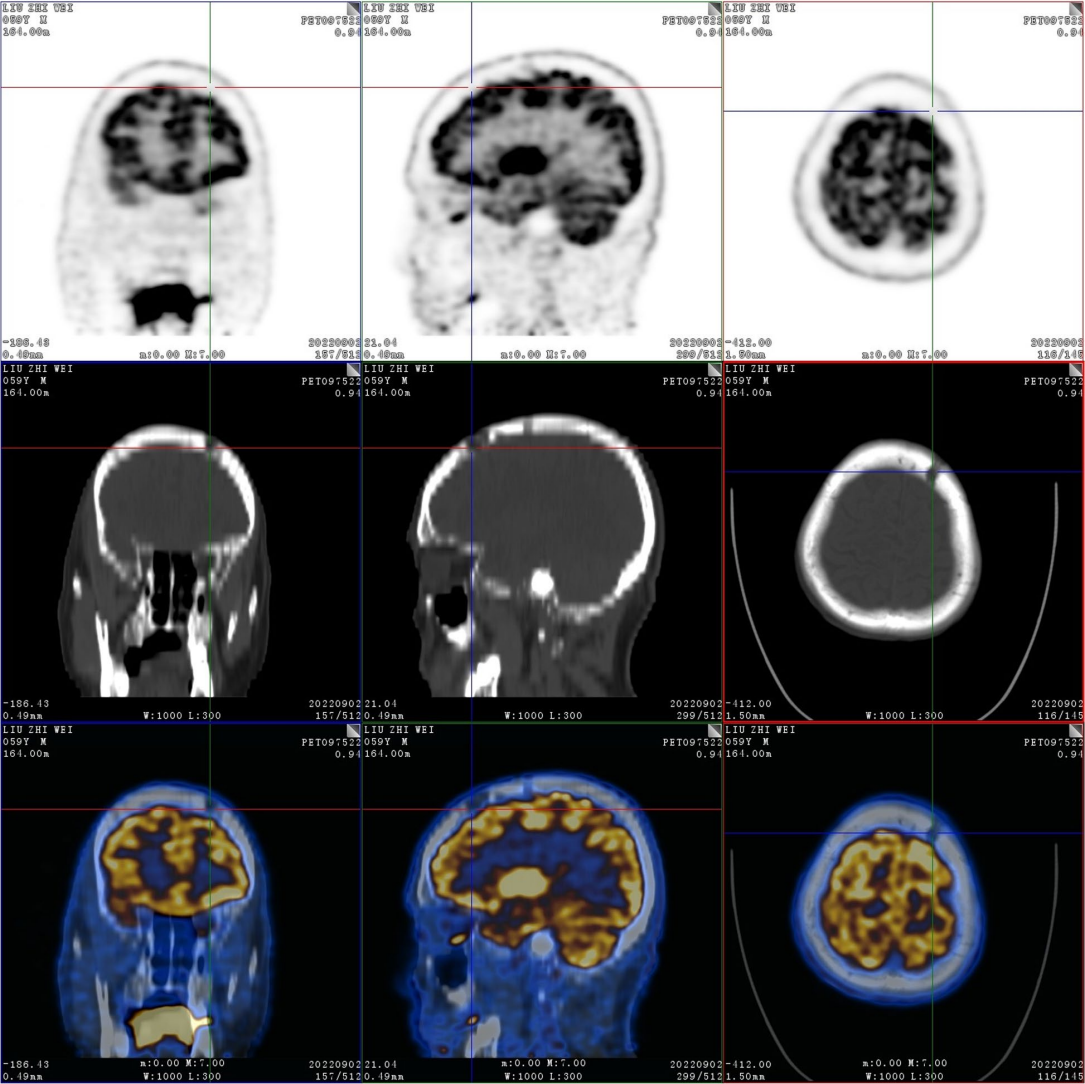
Bone destructions in the left frontoparietal suture

**Fig. 7:**
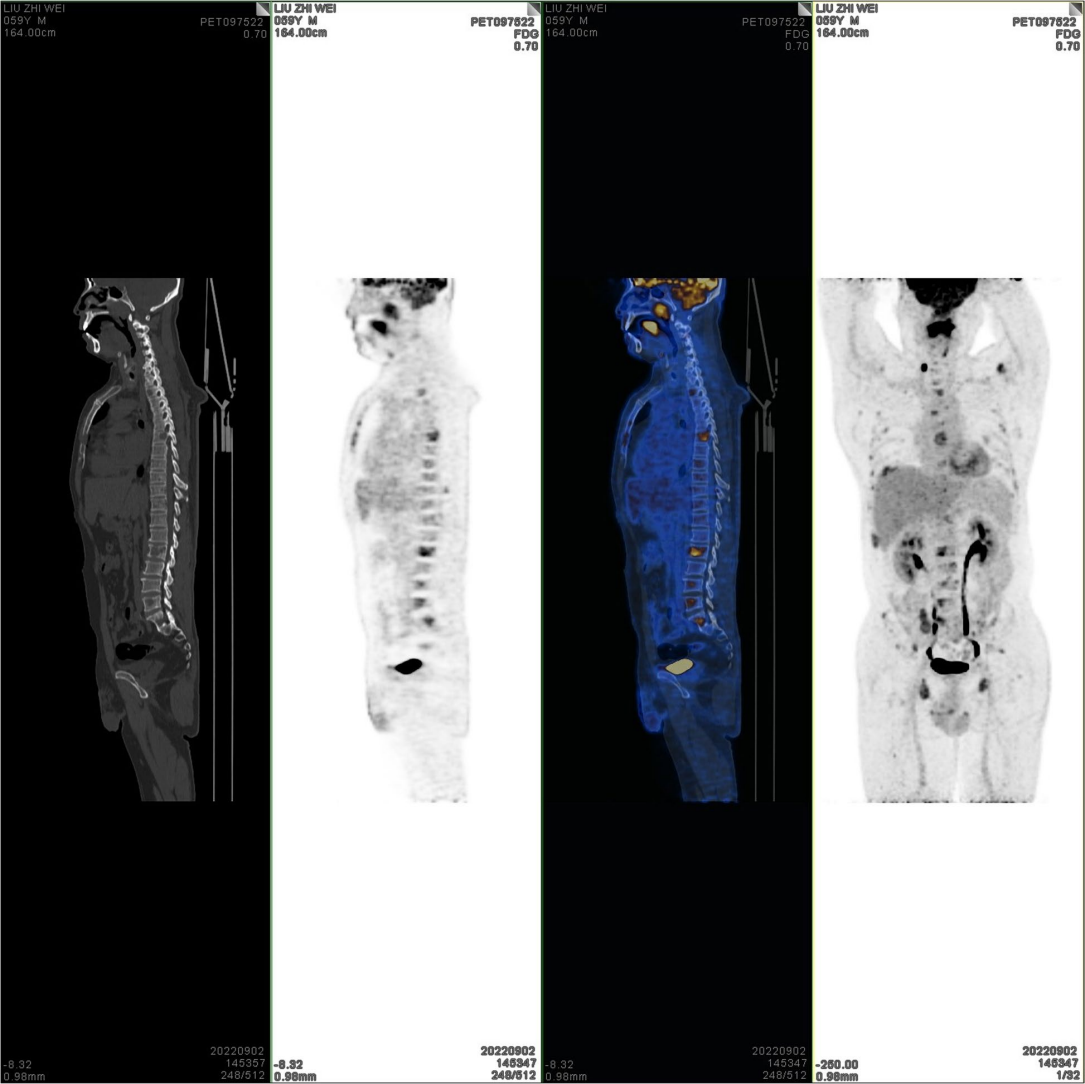
PET-CT scans revealed bone destructions in multiple vertebral bodies and appendix

On September 2, 2022, positron emission tomography-computed tomography (PET-CT) scans revealed the loss of left pharyngeal recess, as well as a palpable mass formed by incrassated soft tissue on posterior and left walls of the nasopharynx, 32 × 30 mm in size, of which the FDG metabolism increased significantly and the SUVmax value was 9.1 (Fig. 1). Diffuse elevation of FDG metabolism was also found in the tongue, SUVmax = 21.4. In addition, PET-CT scans also revealed an abnormality in kidney and thyroids. An isodense nodule was observed in the right superior renal pole, approximately 25 mm in diameter, with an increase in FDG metabolism (Fig. 2). Both lobes of the thyroid were normal in shape and size. However, there was a slightly hypodense shadow in the right leaf of the thyroid, approximately 13 mm in diameter, with an unusually high level of FDG metabolism, SUVmax = 26.5 (Fig. 3). According to the above results, the possibility of synchronous malignant tumors in the right kidney, nasopharynx, and right lobe of thyroid was suggested. Other lesions detected include a nodule in the inferior lobe of the left lung which was 12 × 10 mm in size (Fig. 4), and multiple patchy shadows with low density in the liver (Fig. 5). Last but not least, multiple bone destructions were revealed in the left frontoparietal suture (Fig. 6), multiple vertebral bodies and appendix (Fig. 7), cricoid cartilage, bilateral scapulae, multiple bilateral ribs, pelvis bones and bilateral femurs, accompanied with increased FDG metabolism, suggesting a large possibility of systemic bone metastases.

**Fig. 8:**
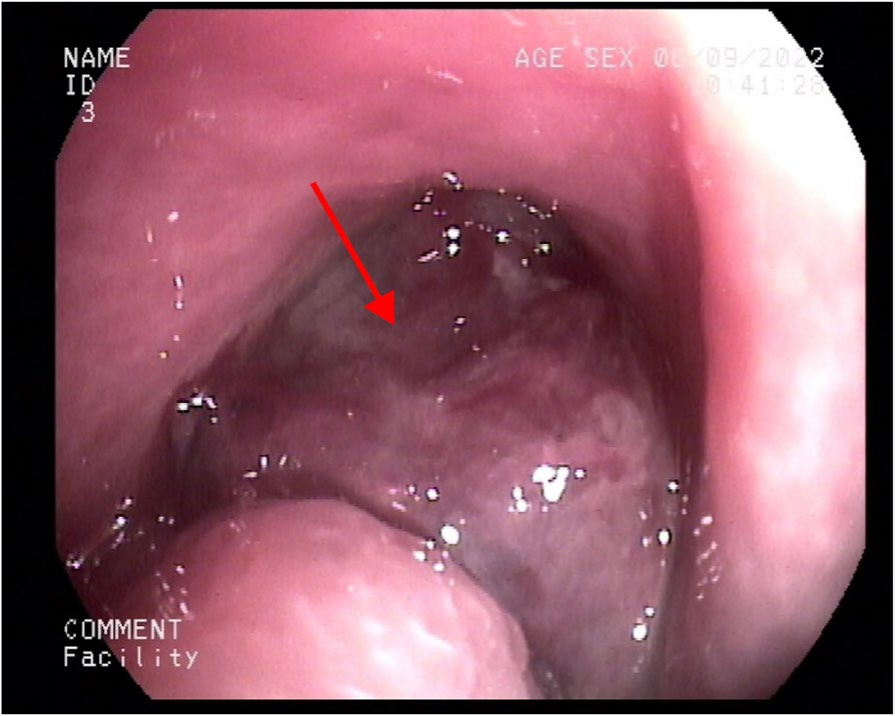
The result of nasal endoscopy. The *arrow* points towards the gigantic tumor-like neoplasm in nasopharynx

**Fig. 9:**
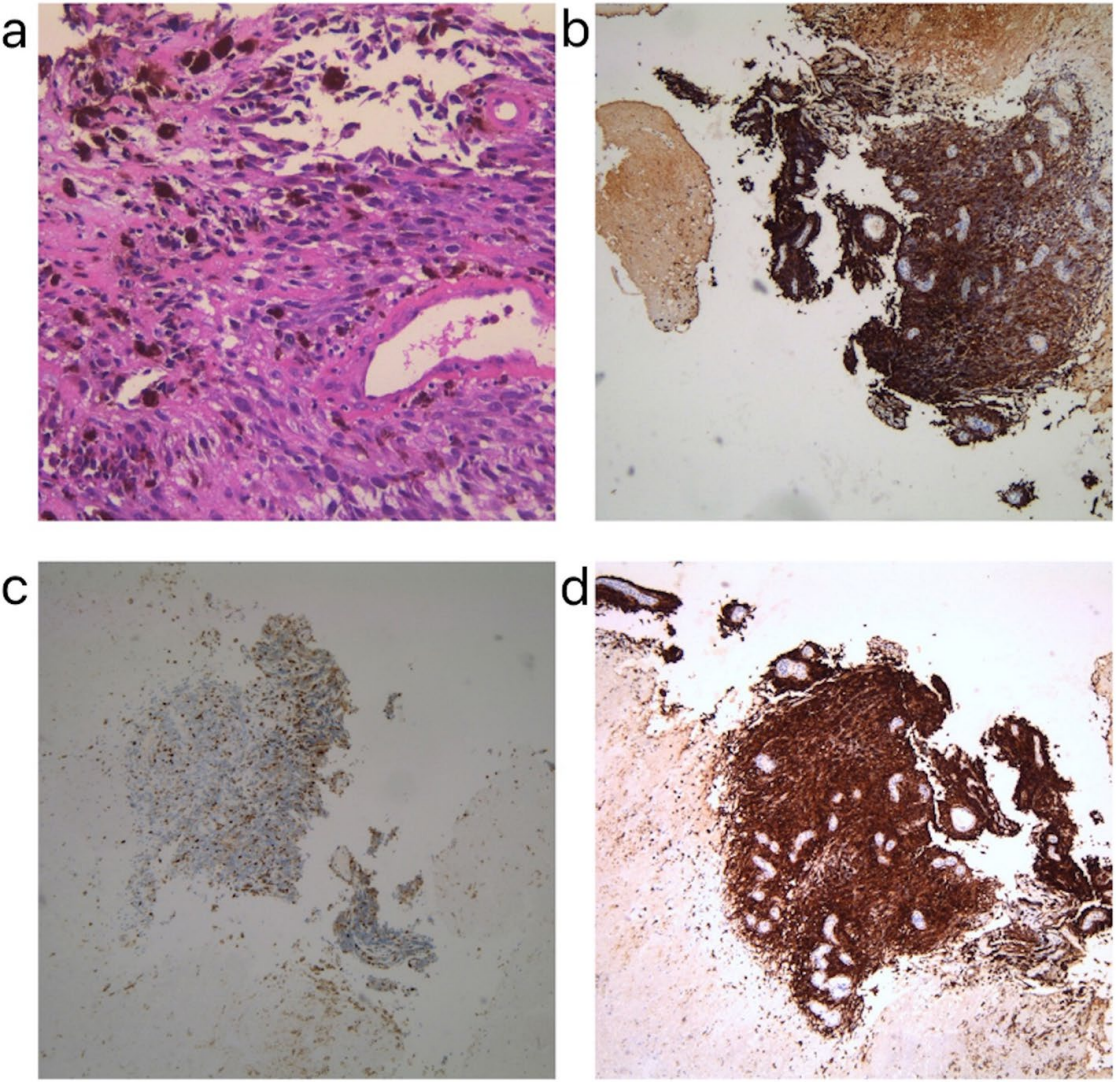
**a** Pathological results of the neoplasm in nasopharynx. Haematoxylin–eosin (H&E) staining of biopsy samples (× 100) magnification. **b** Immunohistochemical staining results of the neoplasm in nasopharynx showed HMB45 ( +) **c** Ki67 (10%) **d** MelanA ( +)

On September 6, 2022, nasal endoscopy revealed a gigantic tumor-like neoplasm in the nasopharynx and hyperplastic lymphoid follicles in the posterior pharyngeal wall and root of the tongue (Fig. 8). Mild hyperemia of the pharyngeal mucosa was also observed. On September 7, 2022, a nasopharynx enhanced magnetic resonance imaging (MRI) scan revealed a distinct incrassation of the nasopharyngeal posterior wall; and an irregular-shaped mass shadow with slightly hyperintense on T1W1 and hypointense on T2W1 was be observed, 33 × 27 mm in size. After injecting the contrast agent, the scan showed significant enhancement in the mass, as well as loss of bilateral pharyngeal recesses and the narrowing of nasopharyngeal cavity. In addition, multiple vertebral bodies demonstrated variable signal intensity on MR scans, and showed significant enhancement following contrast administration. There were also patchy shadows in the left mastoid region with slightly hypointense on T1W1 and hyperintense on T2W1. On September 19, 2022, the pathological results of the nasopharyngeal biopsy (Fig. 9) revealed that around most necrotic tissues in the tumor-like neoplasm there were a small amount of heterocysts, accompanied with hemosiderin deposition. On September 26, 2022, the final results of biopsy immunohistochemistry (Fig. 9) showed the following: CAM5.2 ( −), VIM ( +), P53 ( −), Ki67 (10%), SOX10 ( +), SY ( −), CD56 ( −), CD20 ( −), CgA ( −), CD31 ( −), Desmin ( −), P40 ( −), HMB45 ( +), MelanA ( +). Gene detection showed amplification of CCND1 and mutation of BRAF oncogene. In combination with the pathological results, it was suggested that there was a nasopharyngeal malignant melanoma. On September 15, 2022, immunohistochemical results of intercostal puncture showed the following: VIM ( +), S-100 ( +), HMB45 ( +), MelanA ( +), PD-L1/22C3 (3%), Ki67 (40%), CAM5.2 ( −). Gene detection showed a mutation in MSS, SF3B1, Ep300, MYC, CCND1, and PAK1. And therefore, bone metastasis of malignant melanoma was suggested according to the above results.

On September 9, 2022, a color-Doppler guided fine-needle aspiration biopsy of the thyroid gland was performed. The result revealed a hypoechoic nodule with microcalcification (TI-RADS 4a) in the lower part of left lobe of the thyroid, 5 × 4.8 mm in size, and multiple nodules (TI-RADS 4b) in both upper and lower part of the right lobe of thyroid, respectively 12.5 × 10.3 mm and 6.7 × 4.6 mm in size. On September 12, 2022, the genetic test results of thyroid needle biopsy showed a mutation of BRAF^V600E^ in both the left and right lobes of thyroid. The pathological results subsequently revealed papillary carcinoma (Bethesda category VI) in both lesions of the left and right lobe of thyroid.

On September 15, 2022, the patient underwent a renal needle biopsy, of which the pathological results revealed clear cell carcinoma and the immunohistochemistry results showed the following: PAX-8 ( +), CAIX ( +). Gene detection showed that the tumor mutation burden (TMB) was 16.58 Muts/Mb. The morphological and immunohistochemical results were consistent with a clear-cell renal cell carcinoma (ccRCC).

### Treatment

The patient was transferred to Fudan University Shanghai Cancer Center on September 30, 2022. After excluding contraindications of chemotherapy, Cisplatin 40 mg ivggt d1-d3 + Toripalimab 240 mg ivggt d1 + Endostar 180 mg civ72h d1 + Temozolomide 200 mg op d1–d5 once/3 weeks were prescribed for treatment. At present, the patient remains stable and his overall condition is good. No adverse reaction such as marrow suppression has developed yet.

## Discussion and conclusions

Patients with malignant melanoma are at the risk of developing second primary tumors. According to a study by Bhatia et al. (1999), among the total population of 585 malignant melanoma patients, 37 patients (6.3%) were diagnosed with a second primary tumor, of whom 23 had non-melanoma skin cancers and the remainder had Hodgkin’s lymphoma, non-Hodgkin’s lymphoma, and carcinoma of the breast, urinary bladder, lung, prostate, and cervix [6]. However, co-occurrence of papillary thyroid carcinoma in malignant melanoma patients has rarely been reported.

Goggins et al. (2006) reviewed 73,274 MM cases between 1973 and 2000 and found a 2.17-fold increase (*p* < 0.0000001) in the risk of developing thyroid cancer as a second primary tumor [7]. In another study by Oakley et al. (2014), patients with MM had a 2.3-fold increased risk (*P* < 0.001) of being diagnosed as having PTC compared with population-based matched controls; patients with PTC had a 1.8-fold increased risk (*P* < 0.001) of developing melanoma [8]. Kim et al. (2010) also reported a case of malignant melanoma with the concurrence of papillary thyroid carcinoma [9], suggesting that aggressive forms of melanoma are associated with an increased risk of developing thyroid malignancies.

A genetic link may exist between MM and PTC due to the common vulnerability to BRAF^V600E^ mutagenesis, and therefore can lead to their co-occurrence. It has been reported that patients with MM and PTC show a higher frequency of BRAF mutation compared with patients who are suffering from other types of cancer [10–13]. BRAF mutations are observed in 36 to 83% of cases of PTC in all age groups [14] and in 63% of MM cases [15].

A study by Zerfaoui et al. (2021) explained the link between melanoma and thyroid cancer from the angle of dysregulation of the nucleocytoplasmic trafficking [16]. Involved in diverse cellular functions, the mitogen-activated protein kinase (MAPK, also known as ERK) cascade is a central signal transduction pathway that is activated by growth factors [17–24]. In response to extracellular stimuli, three classes of protein kinases, MAP kinase kinase kinase (MAPKKK), MAP kinase kinase (MAPKK, also known as MEK) and MAPK, are sequentially activated. The activation is initiated by RAS proteins, which are binary molecular switches that cycle between active guanosine triphosphate (GTP)-bound and inactive guanosine diphosphate (GDP)-bound states. RAS–GTP dimers subsequently recruit MAPKKKs or MAPKKK/MEK heterodimers to membranes, where tetramers consisting of MAPKKK and MEK promote MAPKKK activation. MEK activation is initiated by docking on MAPKKK dimers, which further facilitate MAPK phosphorylation [25]. MAPK, which localizes to the cytoplasm in quiescent cells, translocate from the cytoplasm to the nucleus after being activated, where MAPK phosphorylates and activates several nuclear targets such as transcription factors [26–28]. However, nuclear localization of MAPK is transient and MAPK soon relocalizes to the cytoplasm again to prepare for the next activation. Nucleocytoplasmic trafficking is the transport of proteins, RNAs and signaling molecules between the nucleus and the cytoplasm [29]. Nucleocytoplasmic transport of proteins is achieved when nuclear localization sequences (NLS) and nuclear export sequences (NES) on the cargo protein form a complex with importin or exportin, and the cargo-receptor complex then bind to nucleoporins via the receptor. CRM1 is a main exportin which can phosphorylate MAPK/ERK kinases (MEKs) by shuttling BRAF proteins into and out of the nucleus. Zerfaoui et al. (2021) pointed out that mutation of BRAF activates CRM1 and can lead to dysregulation of the RAS-RAF-MEK-ERK pathway, thus causing continuous nuclear translocation of ERK and other signaling molecules, which is the major common cause in the development of MM and PTC [16]. Moreover, the BRAF mutation is almost exclusively a thymine-to-adenine transversion at position 1799, leading to a valine-to-glutamate substitution at residue 600 (V600E) [10]. Cohen et al. (2003) also indicated that the BRAF T1799A mutation can lead to activation at various points in the RAS-RAF-MEK-ERK pathway, which is a major mechanism in the most common type of malignant thyroid tumor [12]. However, by blocking CRM1, the nuclear export of ERK is inhibited because the relocalization of nuclear ERK to the cytoplasm involves MEK1, which contains the NES sequence [30]. And therefore, the inhibition of the activity of CRM1 has been explored as an important therapeutic target in melanoma and thyroid cancers.

In addition, the V600E mutation of BRAF is associated with microsatellite instability (MSI). Microsatellite instability (MSI) and mismatch repair deficiency are an emerging issue in oncology and molecular pathology. Strong evidence shows that MSI is a frequent event in melanoma [31]. However, the data on MSI prevalence, pathogenesis, and clinical consequences in melanoma are limited. Therefore, this remains to be a potential mechanism.

There are also other theories explaining the link between melanoma and thyroid cancer. A study by Shah et al. (2006) suggests the possibility that hypothyroidism being more frequent among melanoma patients because melanoma tumors may affect the hypothalamus–pituitary–thyroid axis, thus shifting the thyroid stimulating hormone (TSH) levels [32]. On the other hand, since the TSH, which has been reported to be able to convert melanocytes to melanoma, is elevated in patients with thyroid failure and the thyroid stimulating hormone receptors (TSHR) are highly expressed in melanomas, it has been postulated that TSH activates the TSHR signaling pathways, which are critical in the development of melanoma [33, 34]. In melanoma cells, TSHR was proven to be functional, as demonstrated by the ability of TSH to stimulate cAMP formation and the MAPK pathway [34, 35]. In addition, cultured melanoma cells were prompted to proliferate by physiological TSH concentrations. These results clearly indicate a role for TSH in melanoma progression.

Interestingly and likewise, the co-occurrence of MM and renal cell carcinoma (RCC) seems not to be a random result either. In the study by Wu et al. (2006), a total of 955 patients diagnosed with MM between 1987 and 2001 were analyzed, and the results show an increased risk of developing RCC (SIR = 2.41, 95% CI, 0.97–4.97) in male MM patients [36]. It is speculated that this coexistence might be attributed to mutual carcinogenic exposure, aberration of cell-mediated immunity and a shared genetic susceptibility.

Another study also confirmed the potential link between MM and RCC, and most of the RCC cases in this study were clear cell renal cell carcinoma (ccRCC). In addition, compared to only MM and only RCC groups, MM-RCC coexistence group had more family history of cancer, and CDKN2A and CDK4 were found to be predisposing to this association [37]. The main functions of these two genes are to regulate cell cycle, and when a mutation happens, these genes also contribute to tumorigenesis [38].

Cyclin D1, the encoding product of CCND1 which is another gene that regulates the cell cycle, can form active complexes with CDK4, which, in turn, phosphorylate the retinoblastoma (Rb) protein and drive through the restriction point. Mutation in CCND1 can be frequently detected in melanoma [39]. Furthermore, a recent research has reported that the proliferation, migration, and invasion of RCC cells may be promoted by circ-PRKCI via the CCND1 signaling pathway [40]. This result indicates a possible link between the mutation of CCND1 and the co-occurrence of MM and RCC, as for mutations of CCND1 increases phosphorylation of Rb and promotes the drive through restriction, which can result in the release of E2F thus in turn promoting S-phase gene transcription.

In addition to the above discoveries, Bertolotto et al. (2011) found that mutation of the MITF gene, a transcription factor in the Myc oncogene family, might be an inducing factor for the association of MM and RCC [41]. The microphthalmia-associated transcription factor (MITF) has been proposed to act as a melanoma oncogene [42]. In melanocytes, activated MAPK triggers the phosphorylation of MITF at serine-73, recruiting the transcriptional coactivator p300 [43] while simultaneously targeting MITF for ubiquitin-dependent proteolysis [44]. Normal growth and differentiation of melanocyte may also require the synergistic actions of MITF and CDK-inhibitors such as p16 or p21 [45, 46]. Thus, mutation of MITF gene might promote tumour formation through the setting of cell cycle deregulation. On the other hand, MITF oncogene mutation also stimulates the transcription of hypoxia-inducible factor (HIF1A) [47], the pathway of which is targeted by kidney cancer susceptibility genes. Linehan et al. (2010) reported that ccRCC that express HIF1A exhibit enhanced signaling via the MAPK and serine/threonine-protein kinase mammalian target of rapamycin (mTOR) pathways [48].

In this report, we have explored the possible pathogenesis of the co-occurrence of PTC, MM, and ccRCC. Mutation of BRAF^V600E^ was detected in bilateral thyroid tissues, suggesting the possible cause for the co-occurrence of PTC and MM. Furthermore, the result of intercostal puncture showed mutation of both CCND1 and MYC oncogene, and amplification of CCND1 was also detected in the nasopharyngeal melanoma, prompting the possibility that the coexistence of MM and RCC in this case can be explained by the potential genetic link between these two diseases. The BRAF^V600E^ mutation can lead to activation of the RAS-RAF-MEK-ERK pathway, while activated MAPK can subsequently trigger the phosphorylation of MITF, resulting in cell cycle deregulation. Moreover, amplification of CCND1 also promotes the cell cycle through facilitating the drive through restriction. Such linkage among BRAF, CCND1, and MYC mutations may account for the pathogenesis of the coexistence of MM, PTC, and ccRCC. Issues to be addressed in future studies include interactions between environmental exposures and genetic susceptibility and the identification of individuals at increased risk.

## Data Availability

Not applicable.

## Abbreviations

MPMT: Multiple primary malignant tumor
MM: Malignant melanoma
PTC: Papillary thyroid carcinoma
RCC: Renal cell carcinoma
ccRCC: Clear-cell renal cell carcinoma
PET-CT: Positron emission tomography-computed tomography
MRI: Magnetic resonance imaging
TMB: Tumor mutation burden
MAPK/ERK: Mitogen-activated protein kinase
MAPKK/MEK: MAP kinase kinase
MAPKKK: MAP kinase kinase kinase
GTP: Guanosine triphosphate
GDP: Guanosine diphosphate
NLS: Nuclear localization sequences
NES: Nuclear export sequences
MSI: Microsatellite instability
TSH: Thyroid stimulating hormone
TSHR: Thyroid stimulating hormone receptor
Rb: Retinoblastoma
MITF: Microphthalmia-associated transcription factor
mTOR: Mammalian target of rapamycin

## References

[1] Moertel CG, Dockerty MB, Baggenstoss AH (1961). Multiple primary malignant neoplasms. II. Tumors of different tissues or organs. Cancer..

[2] Warren SGO (1932). Multiple primary malignant tumors: A survey of the literature and a statistical study. Am J Cancer.

[3] WHO/IARC (2005). International rules for multiple primary cancers.. Asian Pac J Cancer Prev.

[4] Vogt A, Schmid S, Heinimann K, Frick H, Herrmann C, Cerny T (2017). Multiple primary tumours: challenges and approaches, a review. ESMO Open.

[5] Yamamoto S, Yoshimura K, Ri S, Fujita S, Akasu T, Moriya Y (2006). The risk of multiple primary malignancies with colorectal carcinoma. Dis Colon Rectum.

[6] Bhatia S, Estrada-Batres L, Maryon T, Bogue M, Chu D (1999). Second primary tumors in patients with cutaneous malignant melanoma. Cancer.

[7] Goggins W, Daniels GH, Tsao H (2006). Elevation of thyroid cancer risk among cutaneous melanoma survivors. Int J Cancer.

[8] Oakley GM, Curtin K, Layfield L, Jarboe E, Buchmann LO, Hunt JP (2014). Increased melanoma risk in individuals with papillary thyroid carcinoma. JAMA Otolaryngol Head Neck Surg.

[9] Kim CY, Lee SH, Oh CW (2010). Cutaneous malignant melanoma associated with papillary thyroid cancer. Ann Dermatol.

[10] Davies H, Bignell GR, Cox C, Stephens P, Edkins S, Clegg S (2002). Mutations of the BRAF gene in human cancer. Nature.

[11] Kimura ET, Nikiforova MN, Zhu Z, Knauf JA, Nikiforov YE, Fagin JA (2003). High prevalence of BRAF mutations in thyroid cancer: genetic evidence for constitutive activation of the RET/PTC-RAS-BRAF signaling pathway in papillary thyroid carcinoma. Cancer Res.

[12] Cohen Y, Xing M, Mambo E, Guo Z, Wu G, Trink B (2003). BRAF mutation in papillary thyroid carcinoma. J Natl Cancer Inst.

[13] Xu X, Quiros RM, Gattuso P, Ain KB, Prinz RA (2003). High prevalence of BRAF gene mutation in papillary thyroid carcinomas and thyroid tumor cell lines. Cancer Res.

[14] Tavares C, Melo M, Cameselle-Teijeiro JM, Soares P, Sobrinho-Simões M (2016). ENDOCRINE TUMOURS: Genetic predictors of thyroid cancer outcome. Eur J Endocrinol.

[15] Cicenas J, Tamosaitis L, Kvederaviciute K, Tarvydas R, Staniute G, Kalyan K (2017). KRAS, NRAS and BRAF mutations in colorectal cancer and melanoma. Med Oncol.

[16] Zerfaoui M, Dokunmu TM, Toraih EA, Rezk BM, Abd Elmageed ZY, Kandil E. New insights into the link between melanoma and thyroid cancer: role of nucleocytoplasmic trafficking. Cells. 2021;10(2):367. 10.3390/cells10020367 PMC7916461 33578751

[17] Sturgill TW, Wu J (1991). Recent progress in characterization of protein kinase cascades for phosphorylation of ribosomal protein S6. Biochim Biophys Acta.

[18] Nossal GJ (1992). The molecular and cellular basis of affinity maturation in the antibody response. Cell.

[19] Blenis J (1993). Signal transduction via the MAP kinases: proceed at your own RSK. Proc Natl Acad Sci U S A.

[20] Davis RJ (1993). The mitogen-activated protein kinase signal transduction pathway. J Biol Chem.

[21] Nishida E, Gotoh Y (1993). The MAP kinase cascade is essential for diverse signal transduction pathways. Trends Biochem Sci.

[22] Marshall CJ (1994). MAP kinase kinase kinase, MAP kinase kinase and MAP kinase. Curr Opin Genet Dev.

[23] Lewis TS, Shapiro PS, Ahn NG (1998). Signal transduction through MAP kinase cascades. Adv Cancer Res.

[24] Robinson MJ, Cobb MH (1997). Mitogen-activated protein kinase pathways. Curr Opin Cell Biol.

[25] Li Q, Li Z, Luo T, Shi H (2022). Targeting the PI3K/AKT/mTOR and RAF/MEK/ERK pathways for cancer therapy. Mol Biomed.

[26] Chen RH, Sarnecki C, Blenis J (1992). Nuclear localization and regulation of erk- and rsk-encoded protein kinases. Mol Cell Biol.

[27] Gonzalez FA, Seth A, Raden DL, Bowman DS, Fay FS, Davis RJ (1993). Serum-induced translocation of mitogen-activated protein kinase to the cell surface ruffling membrane and the nucleus. J Cell Biol.

[28] Lenormand P, Sardet C, Pagès G, L'Allemain G, Brunet A, Pouysségur J (1993). Growth factors induce nuclear translocation of MAP kinases (p42mapk and p44mapk) but not of their activator MAP kinase kinase (p45mapkk) in fibroblasts. J Cell Biol.

[29] Macara IG (2001). Transport into and out of the nucleus. Microbiol Mol Biol Rev..

[30] Adachi M, Fukuda M, Nishida E (2000). Nuclear export of MAP kinase (ERK) involves a MAP kinase kinase (MEK)-dependent active transport mechanism. J Cell Biol.

[31] Kubeček O, Kopecký J (2016). Microsatellite instability in melanoma: a comprehensive review. Melanoma Res.

[32] Shah M, Orengo IF, Rosen T (2006). High prevalence of hypothyroidism in male patients with cutaneous melanoma. Dermatol Online J.

[33] Lazzara DR, Zarkhin SG, Rubenstein SN, Glick BP (2019). Melanoma and thyroid carcinoma: our current understanding. J Clin Aesthet Dermatol.

[34] Ellerhorst JA, Sendi-Naderi A, Johnson MK, Cooke CP, Dang SM, Diwan AH (2006). Human melanoma cells express functional receptors for thyroid-stimulating hormone. Endocr Relat Cancer.

[35] Ursu HI (2012). Functional TSH receptors, malignant melanomas and subclinical hypothyroidism. Eur Thyroid J.

[36] Wu YH, Kim GH, Wagner JD, Hood AF, Chuang TY (2006). The association between malignant melanoma and noncutaneous malignancies. Int J Dermatol.

[37] Maubec E, Chaudru V, Mohamdi H, Grange F, Patard JJ, Dalle S (2010). Characteristics of the coexistence of melanoma and renal cell carcinoma. Cancer.

[38] Della Torre G, Pasini B, Frigerio S, Donghi R, Rovini D, Delia D (2001). CDKN2A and CDK4 mutation analysis in Italian melanoma-prone families: functional characterization of a novel CDKN2A germ line mutation. Br J Cancer.

[39] Qie S, Diehl JA (2016). Cyclin D1, cancer progression, and opportunities in cancer treatment. J Mol Med (Berl).

[40] Qian Y, Li Y, Xu L, Chen K, Liu N, Yang X, et al. Tumor cell-derived exosomal circ-PRKCI promotes proliferation of renal cell carcinoma via regulating miR-545–3p/CCND1 axis. Cancers (Basel). 2022;15(1):123. 10.3390/cancers15010123 PMC9817713 36612120

[41] Bertolotto C, Lesueur F, Giuliano S, Strub T, de Lichy M, Bille K (2011). A SUMOylation-defective MITF germline mutation predisposes to melanoma and renal carcinoma. Nature.

[42] Garraway LA, Widlund HR, Rubin MA, Getz G, Berger AJ, Ramaswamy S (2005). Integrative genomic analyses identify MITF as a lineage survival oncogene amplified in malignant melanoma. Nature.

[43] Price ER, Ding HF, Badalian T, Bhattacharya S, Takemoto C, Yao TP (1998). Lineage-specific signaling in melanocytes. C-kit stimulation recruits p300/CBP to microphthalmia. J Biol Chem..

[44] Wu M, Hemesath TJ, Takemoto CM, Horstmann MA, Wells AG, Price ER (2000). c-Kit triggers dual phosphorylations, which couple activation and degradation of the essential melanocyte factor Mi. Genes Dev.

[45] Loercher AE, Tank EM, Delston RB, Harbour JW (2005). MITF links differentiation with cell cycle arrest in melanocytes by transcriptional activation of INK4A. J Cell Biol.

[46] Carreira S, Goodall J, Aksan I, La Rocca SA, Galibert MD, Denat L (2005). Mitf cooperates with Rb1 and activates p21Cip1 expression to regulate cell cycle progression. Nature.

[47] Cheli Y, Ohanna M, Ballotti R, Bertolotto C (2010). Fifteen-year quest for microphthalmia-associated transcription factor target genes. Pigment Cell Melanoma Res.

[48] Linehan WM, Srinivasan R, Schmidt LS (2010). The genetic basis of kidney cancer: a metabolic disease. Nat Rev Urol.

